# Supplementary material for: Lipopolysaccharide stimulates dynamic changes in B cell metabolism to promote proliferation
Source: eLife. 2026 May 21;14:RP109093. doi: 10.7554/eLife.109093 (PMC13193715; doi:10.7554/eLife.109093)
Supplement: Figure 9—figure supplement 1—source data 1. [file elife-109093-fig9-figsupp1-data1.zip › Figure 9 - figure supplement 1 - source data 1/Labelled blot - p38 (top) and p-p38 (bottom).pdf]

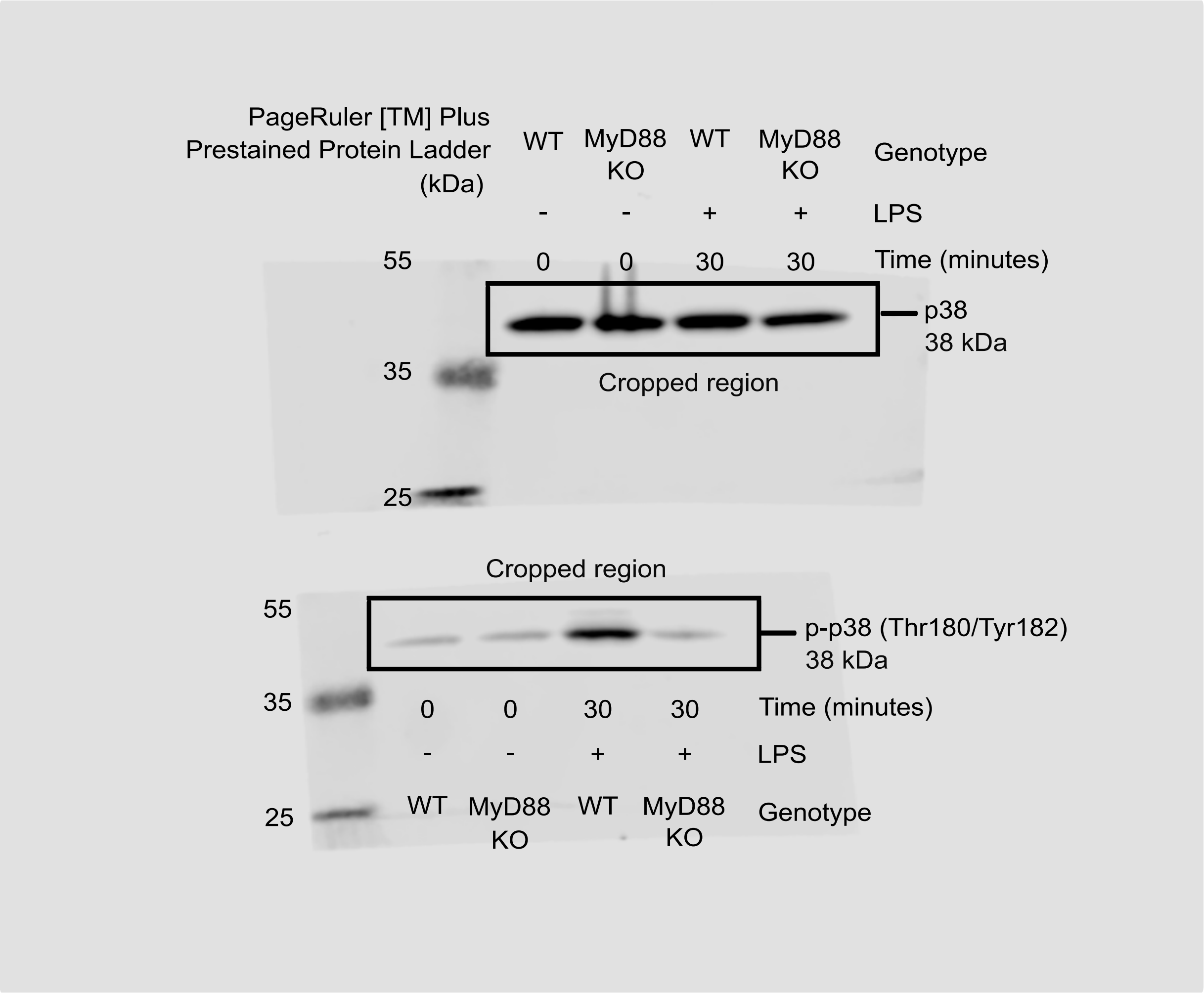


**Figure 9 - figure supplement 1 - source data 1**

Uncropped and labelled membrane corresponding to Figure 9 - figure supplement 1, p38 (top) and p-p38 (Thr180/Tyr182) (bottom).
