# Supplementary figures and images for: Lipopolysaccharide stimulates dynamic changes in B cell metabolism to promote proliferation
Source: eLife. 2026 May 21;14:RP109093. doi: 10.7554/eLife.109093 (PMC13193715; doi:10.7554/eLife.109093)

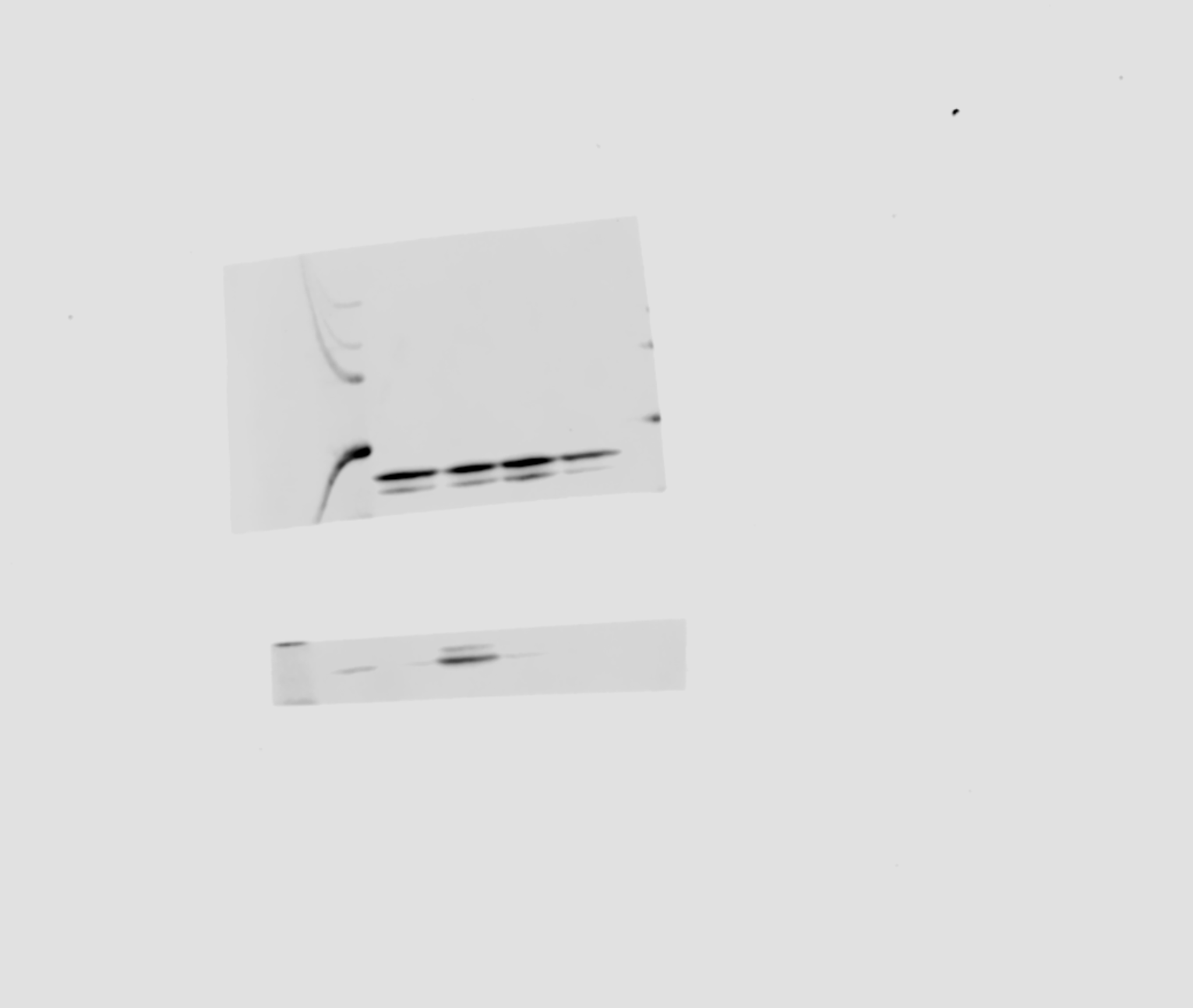

Supplement: Figure 9—figure supplement 1—source data 2. [file elife-109093-fig9-figsupp1-data2.zip › Figure 9 - figure supplement 1 - source data 2/ERK (top) and p-ERK (bottom).tif]

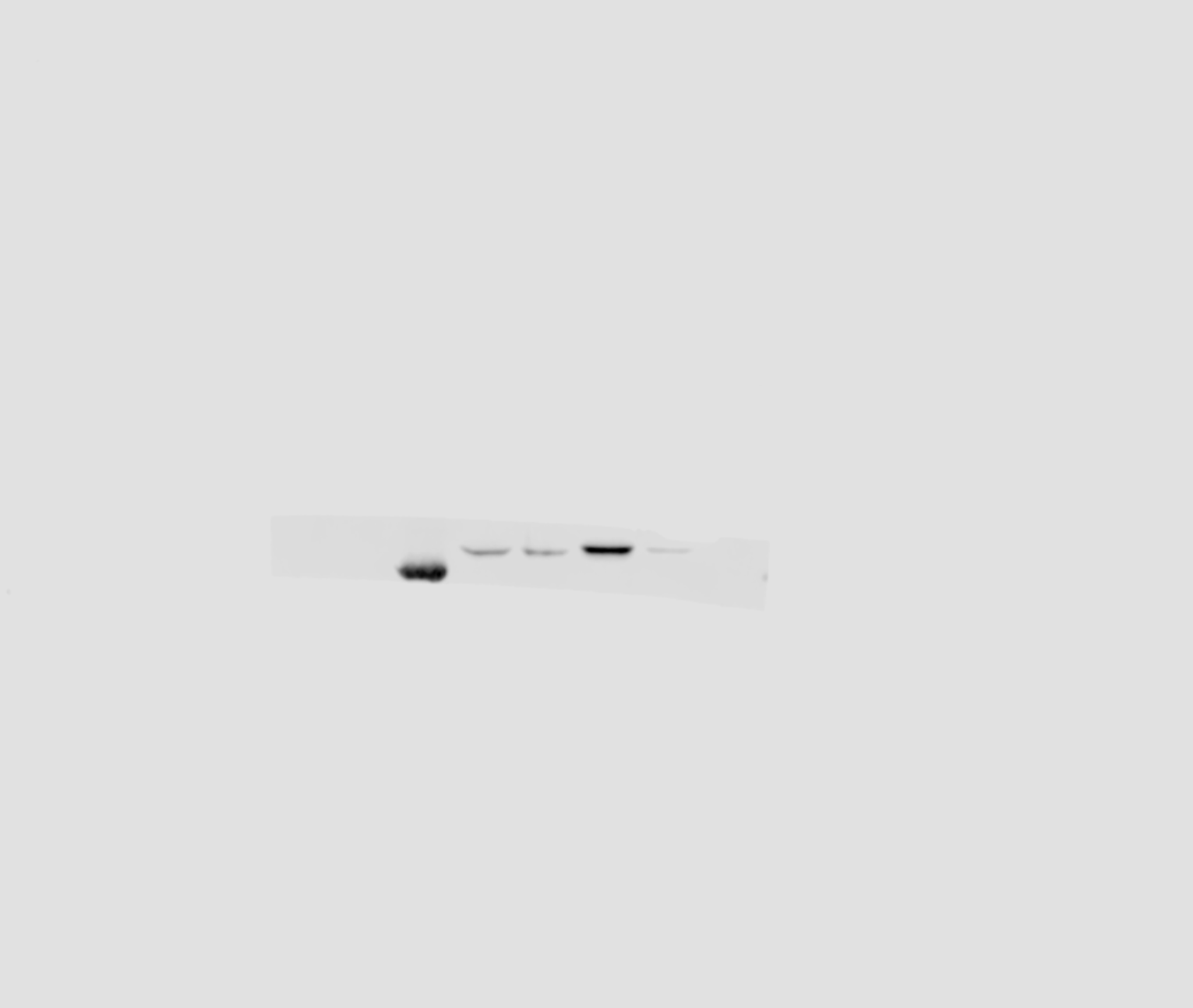

Supplement: Figure 9—figure supplement 1—source data 2. [file elife-109093-fig9-figsupp1-data2.zip › Figure 9 - figure supplement 1 - source data 2/p-AKT.tif]

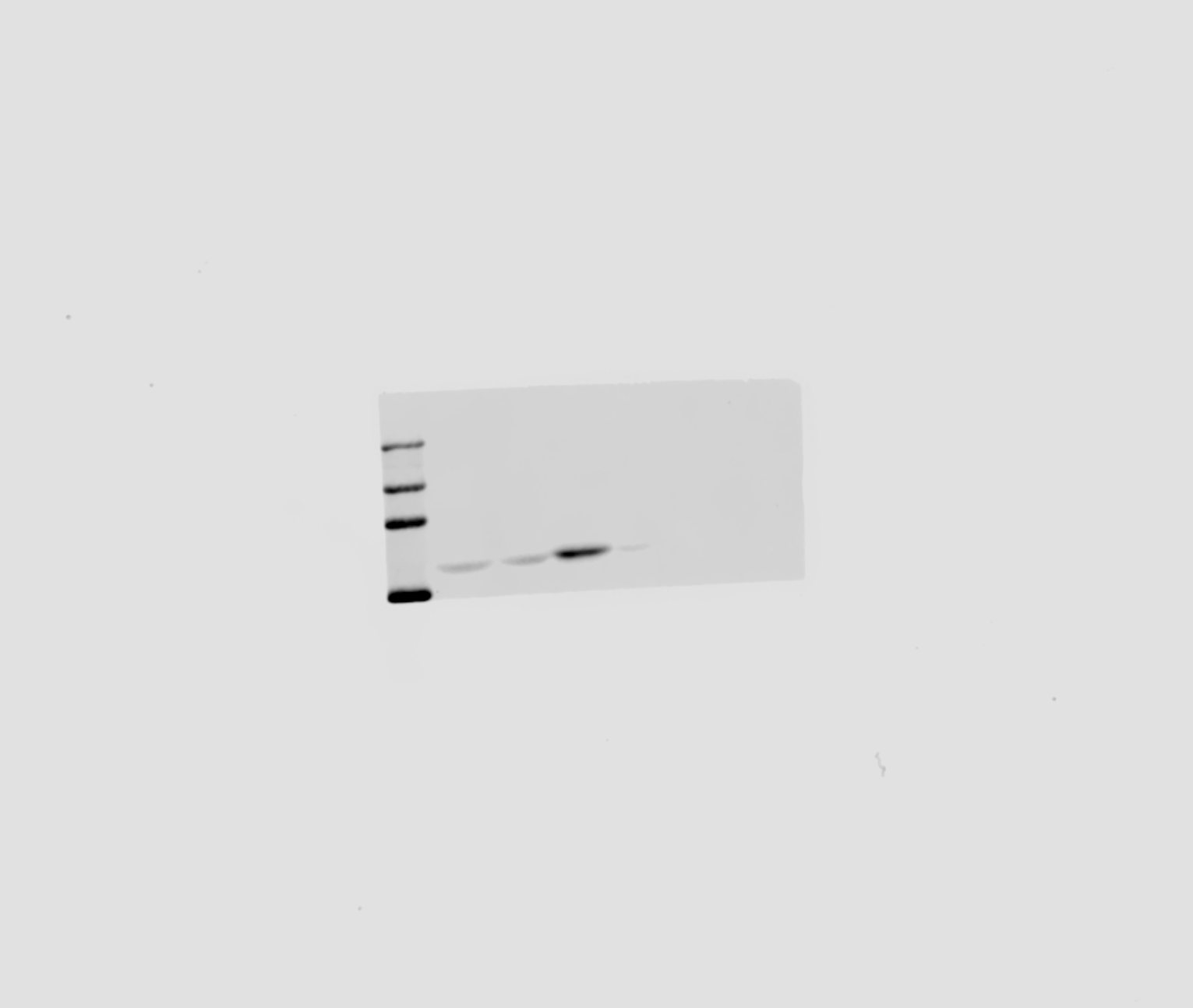

Supplement: Figure 9—figure supplement 1—source data 2. [file elife-109093-fig9-figsupp1-data2.zip › Figure 9 - figure supplement 1 - source data 2/p-P70S6K.tif]

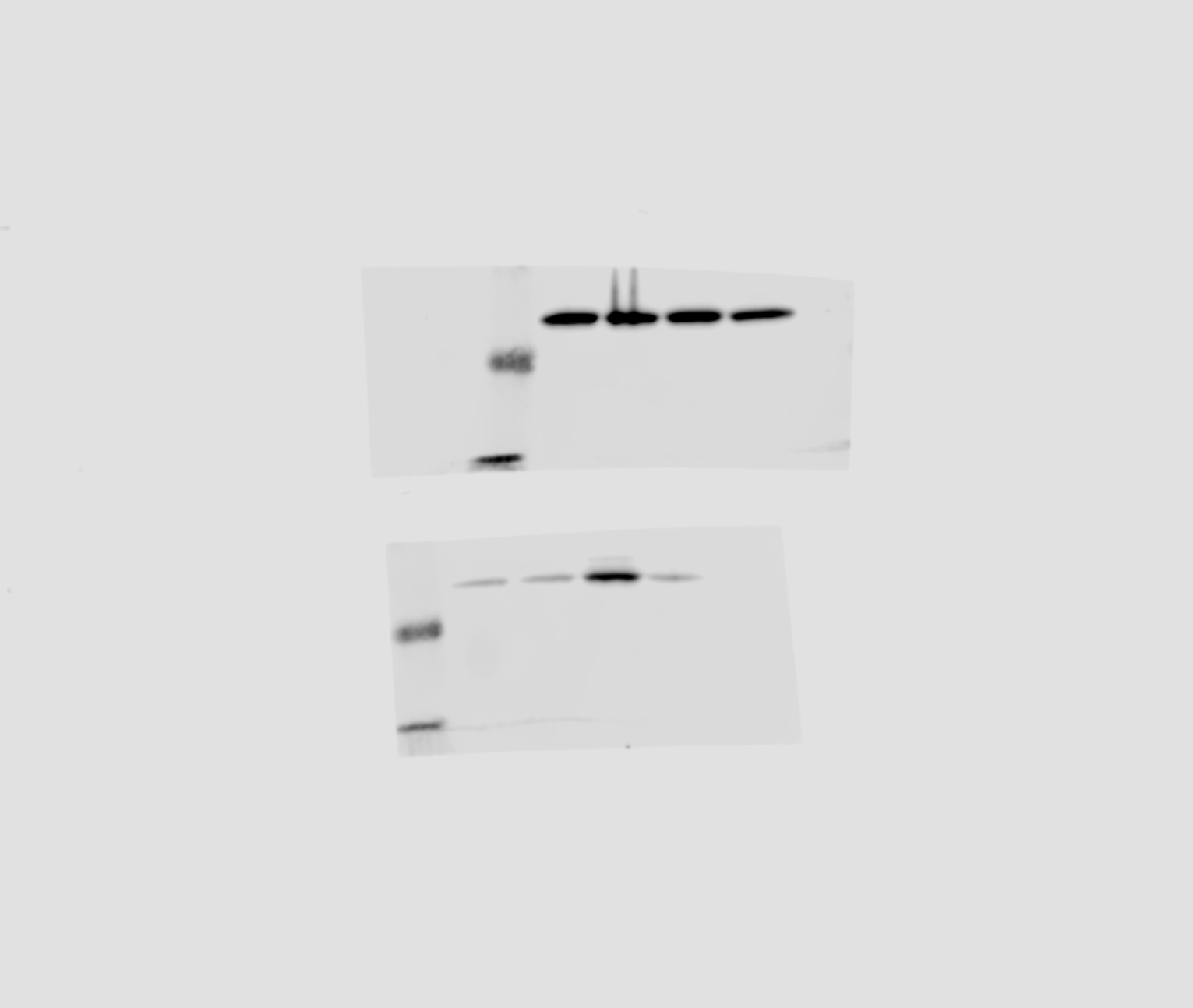

Supplement: Figure 9—figure supplement 1—source data 2. [file elife-109093-fig9-figsupp1-data2.zip › Figure 9 - figure supplement 1 - source data 2/p38 (top) and p-p38 (bottom).tif]

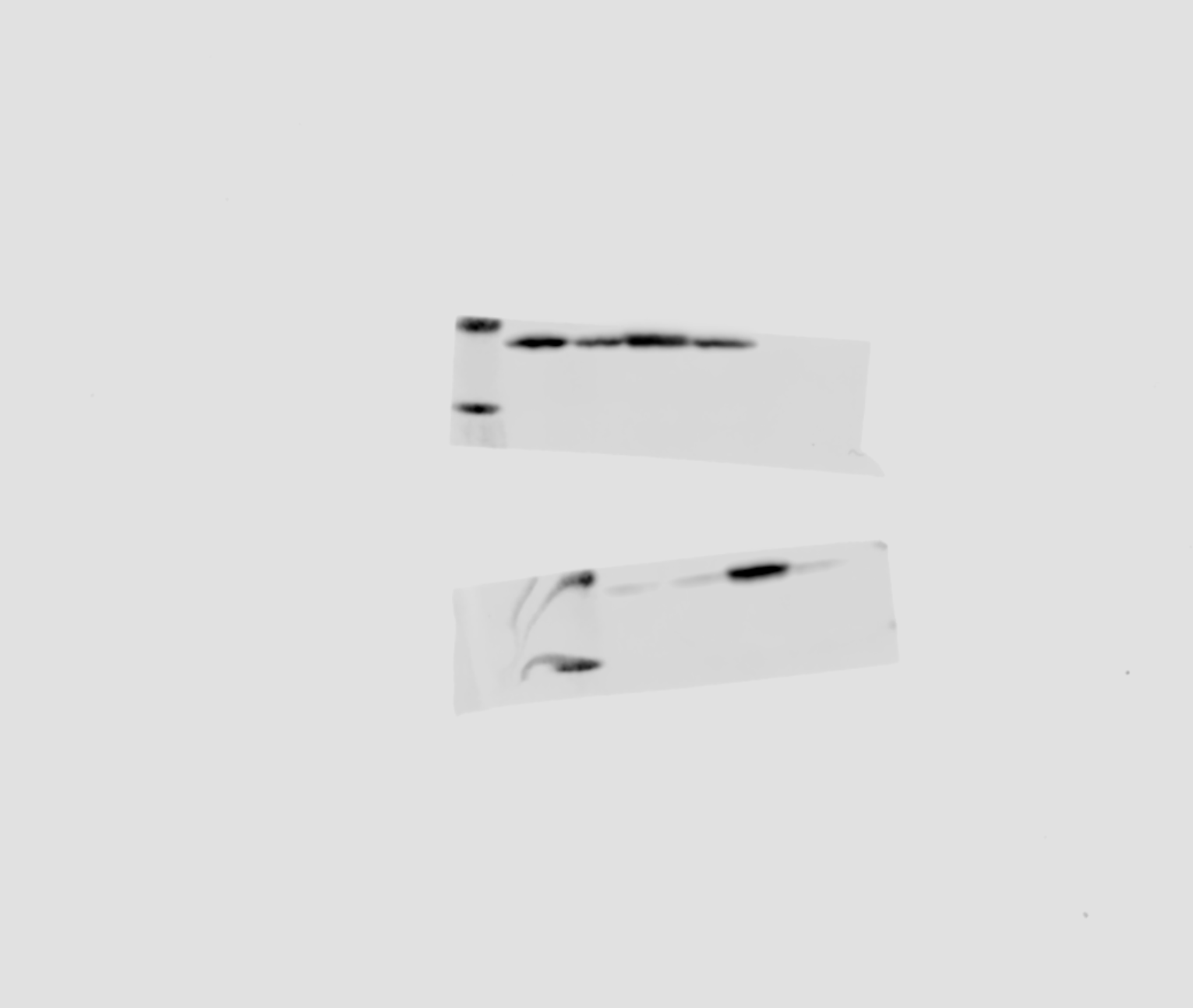

Supplement: Figure 9—figure supplement 1—source data 2. [file elife-109093-fig9-figsupp1-data2.zip › Figure 9 - figure supplement 1 - source data 2/S6 (top) and p-S6 (bottom).tif]
