# Supplementary material for: Lipopolysaccharide stimulates dynamic changes in B cell metabolism to promote proliferation
Source: eLife. 2026 May 21;14:RP109093. doi: 10.7554/eLife.109093 (PMC13193715; doi:10.7554/eLife.109093)
Supplement: Supplementary file 2. [file elife-109093-supp2.docx]

**Supplementary File 2. Serum tests related to Figure 5**

This table presents the results from the Cholesterol/Lipid profile carried out by London Health Company, comparing differences between normal FBS and cholesterol-free FBS.

| **Normal FBS** | **Biochemistry tests** | | | |
| --- | --- | --- | --- | --- |
|  | Test | Result | Normal Range | Units |
|  | **Cholesterol/Lipid Profile** |  |  |  |
|  | Triglycerides | 0.73 | <2.3 | mmol/L |
|  | Cholesterol | 1 | Optimum <5.0 | mmol/L |
|  | HDL Cholesterol | L 0.25 | 0.9-1.7 | mmol/L |
|  | LDL Cholesterol | 0.59 | Up to 3.0 | mmol/L |

| **Cholesterol free FBS** | **Biochemistry tests** | | | |
| --- | --- | --- | --- | --- |
|  | Test | Result | Normal Range | Units |
|  | **Cholesterol/Lipid Profile** |  |  |  |
|  | Triglycerides | 0.67 | <2.3 | mmol/L |
|  | Cholesterol | 0.1 | Optimum <5.0 | mmol/L |
|  | HDL Cholesterol | L 0 | 0.9-1.7 | mmol/L |
|  | LDL Cholesterol | 0.02 | Up to 3.0 | mmol/L |
