# Supplementary material for: Lipopolysaccharide stimulates dynamic changes in B cell metabolism to promote proliferation
Source: eLife. 2026 May 21;14:RP109093. doi: 10.7554/eLife.109093 (PMC13193715; doi:10.7554/eLife.109093)
Supplement: Supplementary file 5. [file elife-109093-supp5.docx]

**Supplementary File 5. Statistics table related to all figures**

This table presents the full results of the ANOVA calculations

| Figure | Test | ANOVA  F value | ANOVA  P value | Test for multiple comparisons | Multiple comparisons (Adjusted P value) | Brown Forsythe test P value |
| --- | --- | --- | --- | --- | --- | --- |
| 1C | One-way ANOVA | 1348 | <0.0001 | Sidak’s multiple comparison test | Naïve vs IL-4: <0.0001  Naive vs LPS + IL-4: <0.0001  IL-4 vs LPS + IL-4 = <0.0001 | 0.2662 |
| 1D | One-way ANOVA | 299.2 | <0.0001 | Sidak’s multiple comparison test | Naïve vs IL-4: 0.0002  Naïve vs LPS + IL-4: <0.0001  IL-4 vs LPS + IL-4 = <0.0001 | 0.3455 |

| Figure | Test | ANOVA  F value | ANOVA  P value | Test for multiple comparisons | Multiple comparisons (Adjusted P value) | Brown Forsythe test P value |
| --- | --- | --- | --- | --- | --- | --- |
| 2B | Two-way ANOVA | Interaction: 152.6  Cell cycle phase: 3368  Stimulation: 9.313 | Interaction: <0.0001  Cell cycle phase: <0.0001  Stimulation: 0.0101 | Sidak’s multiple comparison test | G0_G1: Naïve vs LPS + IL-4: <0.0001  S: Naïve vs LPS + IL-4: 0.0001  G2_M: Naïve vs LPS + IL-4: 0.0023 |  |

| Figure | Test | ANOVA  F value | ANOVA  P value | Test for multiple comparisons | Multiple comparisons (Adjusted P value) | Brown Forsythe test P value |
| --- | --- | --- | --- | --- | --- | --- |
| 4E | One-way ANOVA | 1561 | <0.0001 | Dunnett’s multiple comparison’s test | Naïve vs IL-4: 0.0004  Naïve vs LPS + IL-4: <0.0001 | 0.4645 |
| 4J | Two-way ANOVA | Interaction: 106.6  Condition: 340.1  Genotype: 1707 | Interaction: <0.0001  Condition: <0.0001  Genotype: <0.0001 | Sidak’s multiple comparison test | Naïve WT vs LPS + IL-4 WT: <0.0001  Naïve SLC7A5 KO vs LPS + IL-4 SLC7A5 KO: <0.0001  WT vs SLC7A5 KO (Kynurenine):  <0.0001  WT vs SLC7A5 KO (BCH):  >0.9999  WT vs SLC7A5 KO (HBSS):  <0.0001 |  |
| 4K | Two-way ANOVA | Interaction: 108.1  Condition: 384.4  Genotype: 774.9 | Interaction: <0.0001  Condition: <0.0001  Genotype: <0.0001 | Sidak’s multiple comparisons test | Naïve WT vs LPS WT: <0.0001  Naïve SLC7A5 KO vs LPS SLC7A5 KO: <0.0001  WT vs SLC7A5 KO (Kynurenine): <0.0001  WT vs SLC7A5 KO (BCH):  0.9992  WT vs SLC7A5 KO (HBSS):  0.8046 |  |

| Figure | Test | ANOVA  F value | ANOVA  P value | Test for multiple comparisons | Multiple comparisons (Adjusted P value) | Brown Forsythe test P value |
| --- | --- | --- | --- | --- | --- | --- |
| 5F | One-way ANOVA | 932.6 | <0.0001 | Dunnett’s multiple comparison’s test | Naïve vs 2 hours: 0.0970  Naïve vs 4 hours: 0.0181  Naïve vs 8 hours: <0.0001  Naive vs 16 hours: <0.0001  Naive vs 24 hours: <0.0001 | 0.3544 |
| 5G | One-way ANOVA | 166.1 | <0.0001 | Dunnett’s multiple comparison’s test | Naïve vs LPS + IL-4: <0.0001  LPS + IL-4 vs 0.1µM Fluvastatin: <0.0001  LPS + IL-4 vs 1µM Fluvastatin: <0.0001  LPS + IL-4 vs 10µM Fluvastatin: <0.0001  LPS + IL-4 vs 50µM Fluvastatin: <0.0001  LPS + IL-4 vs 100µM Fluvastatin: <0.0001 | 0.4365 |
| 5H | One-way ANOVA | 147.0 | <0.0001 | Dunnett’s multiple comparison’s test | Naïve vs LPS + IL-4: <0.0001  LPS + IL-4 vs 0.1µM NB-598: 0.0003  LPS + IL-4 vs 1µM  NB-598: <0.0001  LPS + IL-4 vs 10µM  NB-598: <0.0001  LPS + IL-4 vs 50µM  NB-598: <0.0001  LPS + IL-4 vs 100µM NB-598: <0.0001 | 0.8640 |
| 5I | Two-way ANOVA | Interaction: 36.95  Media 332.8  Inhibitor: 488.8 | Interaction: <0.0001  Media: <0.0001  Inhibitor: <0.0001 | Tukey’s multiple comparisons test | Normal  Naïve vs LPS + IL-4:  <0.0001  LPS + IL-4 vs NB-598:  <0.0001  LPS + IL-4 vs Fluvastatin: <0.0001  Normal vs Cholesterol-free LPS + IL-4: <0.0001  Cholesterol-free  Naïve vs LPS + IL-4:  <0.0001  LPS + IL-4 vs NB-598:  <0.0001  LPS + IL-4 vs Fluvastatin: <0.0001 |  |

| Figure | Test | ANOVA  F value | ANOVA  P value | Test for multiple comparisons | Multiple comparisons (Adjusted P value) | Brown Forsythe test P value |
| --- | --- | --- | --- | --- | --- | --- |
| 6B | Two-way ANOVA | Interaction: 8.996  Media: 9.466  Inhibitor: 728.5 | Interaction: 0.0010  Media: 0.0072  Inhibitor: <0.0001 | Tukey’s multiple comparisons test | Normal  Naïve vs LPS + IL-4: <0.0001  LPS + IL-4 vs NB-598: <0.0001  LPS + IL-4 vs Fluvastatin: <0.0001  Normal vs Cholesterol-free  LPS + IL-4: <0.0001  Cholesterol-free  Naïve vs LPS + IL-4: <0.0001  LPS + IL-4 vs NB-598: <0.0001  LPS + IL-4 vs Fluvastatin: <0.0001 |  |
| 6C | Two-way ANOVA | Interaction: 4.956  Media: 7.811  Inhibitor: 645.7 | Interaction: 0.0128  Media: 0.0130  Inhibitor: <0.0001 | Tukey’s multiple comparisons test | Normal  Naïve vs LPS + IL-4: 0.3922  LPS + IL-4 vs NB-598: <0.0001  LPS + IL-4 vs Fluvastatin: <0.0001  Normal vs Cholesterol-free  LPS + IL-4: 0.6252  Cholesterol-free  Naïve vs LPS + IL-4: 0.0012  LPS + IL-4 vs NB-598: <0.0001  LPS + IL-4 vs Fluvastatin: <0.0001 |  |
| 6D | Two-way ANOVA | Interaction: 11.74  Media: 1.331  Inhibitor: 76.74 | Interaction: 0.0003  Media: 0.2655  Inhibitor: <0.0001 | Tukey’s multiple comparisons test | Normal  Naïve vs LPS + IL-4: <0.0001  LPS + IL-4 vs NB-598: 0.0388  LPS + IL-4 vs Fluvastatin: 0.0004  Normal vs Cholesterol-free  LPS + IL-4: 0.0003  Cholesterol-free  Naïve vs LPS + IL-4: <0.0001  LPS + IL-4 vs NB-598: <0.0001  LPS + IL-4 vs Fluvastatin: <0.0001 |  |
| 6F | Two-way ANOVA | Interaction: 14.47  Media: 10.13  Inhibitor: 716.6 | Interaction: 0.0006  Media: 0.0079  Inhibitor: <0.0001 | Tukey’s multiple comparisons test | Normal  Naïve vs LPS + IL-4: <0.0001  LPS + IL-4 vs FGTI-2734: <0.0001  Normal vs Cholesterol-free  LPS + IL-4: <0.0001  Cholesterol-free  Naïve vs LPS + IL-4: <0.0001  LPS + IL-4 vs FGTI-2734: <0.0001 |  |
| 6G | Two-way ANOVA | Interaction: 108.8  Media: 297.7  Inhibitor: 6586 | Interaction: <0.0001  Media: <0.0001  Inhibitor: <0.0001 | Tukey’s multiple comparisons test | Normal  Naïve vs LPS + IL-4: 0.6468  LPS + IL-4 vs FGTI-2734: <0.0001  Normal vs Cholesterol-free  LPS + IL-4: 0.0001  Cholesterol-free  Naïve vs LPS + IL-4: 0.0037  LPS + IL-4 vs FGTI-2734: <0.0001 |  |
| 6H | Two-way ANOVA | Interaction: 42.17  Media: 10.39  Inhibitor: 422.7 | Interaction: <0.0001  Media: 0.0073  Inhibitor: <0.0001 | Tukey’s multiple comparisons test | Normal  Naïve vs LPS + IL-4: <0.0001  LPS + IL-4 vs FGTI-2734: <0.0001  Normal vs Cholesterol-free  LPS + IL-4: <0.0001  Cholesterol-free  Naïve vs LPS + IL-4: <0.0001  LPS + IL-4 vs FGTI-2734: <0.0001 |  |
| 6J | One-way ANOVA | 33.93 | <0.0001 | Dunnett’s multiple comparison’s test | Normal  Naïve vs LPS + IL-4: <0.0001  LPS + IL-4 vs 10µM FTI-277: <0.0001  LPS + IL-4 vs 30µM FTI-277: <0.0001  LPS + IL-4 vs 10µM GGTI-298: <0.0001  LPS + IL-4 vs 30µM GGTI-298: <0.0001 | 0.1288 |
| 6K | One-way ANOVA | 139.2 | <0.0001 | Dunnett’s multiple comparison’s test | Normal  Naïve vs LPS + IL-4: 0.9988  LPS + IL-4 vs 10µM FTI-277: 0.0021  LPS + IL-4 vs 30µM FTI-277: <0.0001  LPS + IL-4 vs 10µM GGTI-298: <0.0001  LPS + IL-4 vs 30µM GGTI-298: <0.0001 | 0.3614 |
| 6L | One-way ANOVA | 66.44 | <0.0001 | Dunnett’s multiple comparison’s test | Normal  Naïve vs LPS + IL-4: <0.0001  LPS + IL-4 vs 10µM FTI-277: 0.0010  LPS + IL-4 vs 30µM FTI-277: <0.0001  LPS + IL-4 vs 10µM GGTI-298: <0.0001  LPS + IL-4 vs 30µM GGTI-298: <0.0001 | 0.47 |
| 6N | One-way ANOVA | 142.3 | <0.0001 | Dunnett’s multiple comparison’s test | CF  Naïve vs LPS + IL-4: <0.0001  LPS + IL-4 vs 10µM FTI-277: <0.0001  LPS + IL-4 vs 30µM FTI-277: <0.0001  LPS + IL-4 vs 10µM GGTI-298: <0.0001  LPS + IL-4 vs 30µM GGTI-298: <0.0001 | 0.3492 |
| 6O | One-way ANOVA | 685.5 | <0.0001 | Dunnett’s multiple comparison’s test | CF  Naïve vs LPS + IL-4: 0.1736  LPS + IL-4 vs 10µM FTI-277: 0.0790  LPS + IL-4 vs 30µM FTI-277: <0.0001  LPS + IL-4 vs 10µM GGTI-298: <0.0001  LPS + IL-4 vs 30µM GGTI-298: <0.0001 | 0.6699 |
| 6P | One-way ANOVA | 1591 | <0.0001 | Dunnett’s multiple comparison’s test | CF  Naïve vs LPS + IL-4: <0.0001  LPS + IL-4 vs 10µM FTI-277: <0.0001  LPS + IL-4 vs 30µM FTI-277: <0.0001  LPS + IL-4 vs 10µM GGTI-298: <0.0001  LPS + IL-4 vs 30µM GGTI-298: <0.0001 | 0.4074 |

| Figure | Test | ANOVA  F value | ANOVA  P value | Test for multiple comparisons | Multiple comparisons (Adjusted P value) | Brown Forsythe test P value |
| --- | --- | --- | --- | --- | --- | --- |
| 7A | Two-way ANOVA | Interaction: 10.82  Time: 27.12  Treatment: 38.02 | Interaction: 0.0021  Time: 0.0002  Treatment: <0.0001 | Sidak’s multiple comparisons test | Normal  LPS + IL-4 vs Fluvastatin (24 hours): 0.0209  Fluvastatin vs MVA (24 hours): 0.8260  LPS + IL-4 vs Fluvastatin (48 hours): <0.0001  Fluvastatin vs MVA (48 hours): <0.0001 |  |
| 7B | Two-way ANOVA | Interaction: 15.32  Time: 14.26  Treatment: 195.1 | Interaction: 0.0005  Time: 0.0026  Treatment: <0.0001 | Sidak’s multiple comparisons test | CF  LPS + IL-4 vs Fluvastatin (24 hours): <0.0001  Fluvastatin vs MVA (24 hours): 0.8756  LPS + IL-4 vs Fluvastatin (48 hours): <0.0001  Fluvastatin vs MVA (48 hours): 0.2388 |  |
| 7C | Two-way ANOVA | Interaction: 302.4  Media: 270.4  Treatment: 922.5 | Interaction: <0.0001  Media: <0.0001  Treatment: <0.0001 | Sidak’s multiple comparisons test | Normal  LPS + IL-4 vs Fluvastatin: <0.0001  Fluvastatin vs MVA: <0.0001  Cholesterol free  LPS + IL-4 vs Fluvastatin: <0.0001  Fluvastatin vs MVA: 0.0023 |  |
| 7D | Two-way ANOVA | Interaction: 18.29  Media: 0.5922  Treatment: 20.81 | Interaction: 0.0002  Media: 0.4565  Treatment: 0.0001 | Sidak’s multiple comparisons test | Normal  LPS + IL-4 vs Fluvastatin: 0.0129  Fluvastatin vs MVA: 0.0009  Cholesterol free  LPS + IL-4 vs Fluvastatin: 0.0006  Fluvastatin vs MVA: 0.7214 |  |
| 7F | Two-way ANOVA | Interaction: 93.12  Media: 0.02330  Treatment: 2594 | Interaction: <0.0001  Media: 0.8812  Treatment: <0.0001 | Sidak’s multiple comparisons test | Normal  LPS + IL-4 vs Fluvastatin: <0.0001  Fluvastatin vs MVA: <0.0001  Cholesterol free  LPS + IL-4 vs Fluvastatin: <0.0001  Fluvastatin vs MVA: 0.9810 |  |

| Figure | Test | ANOVA  F value | ANOVA  P value | Test for multiple comparisons | Multiple comparisons (Adjusted P value) | Brown Forsythe test P value |
| --- | --- | --- | --- | --- | --- | --- |
| 8B | One-way ANOVA on log transformed data | 37.49 | <0.0001 | Dunnett’s multiple comparison’s test | Fluvastatin vs LPS + IL-4: <0.0001  Fluvastatin vs MVA: 0.0003  Fluvastatin vs GGPP: <0.0001  Fluvastatin vs MVA + GGPP: <0.0001 | 0.9972 |
| 8C | One-way ANOVA | 63.63 | <0.0001 | Dunnett’s multiple comparison’s test | Fluvastatin vs LPS + IL-4: <0.0001  Fluvastatin vs MVA: <0.0001  Fluvastatin vs GGPP: <0.0001  Fluvastatin vs MVA + GGPP: <0.0001 | 0.7134 |
| 8D | One-way ANOVA | 52.03 | <0.0001 | Dunnett’s multiple comparison’s test | Fluvastatin vs LPS + IL-4: <0.0001  Fluvastatin vs MVA: <0.0001  Fluvastatin vs GGPP: <0.0001  Fluvastatin vs MVA + GGPP: <0.0001 | 0.8877 |
| 8F | One-way ANOVA on log transformed data | 38.63 | <0.0001 | Dunnett’s multiple comparison’s test | Fluvastatin vs LPS + IL-4: <0.0001  Fluvastatin vs MVA: 0.248  Fluvastatin vs GGPP: 0.4568  Fluvastatin vs MVA + GGPP: 0.9404 | 0.9069 |
| 8G | One-way ANOVA | 3461 | <0.0001 | Dunnett’s multiple comparison’s test | Fluvastatin vs LPS + IL-4: <0.0001  Fluvastatin vs MVA: <0.0001  Fluvastatin vs GGPP: <0.0001  Fluvastatin vs MVA + GGPP: <0.0001 | 0.8872 |
| 8H | One-way ANOVA | 23.61 | <0.0001 | Dunnett’s multiple comparison’s test | Fluvastatin vs LPS + IL-4: 0.0005  Fluvastatin vs MVA: 0.0893  Fluvastatin vs GGPP: 0.1305  Fluvastatin vs MVA + GGPP: 0.9897 | 0.7828 |
| 8I | Two-way ANOVA | Interaction: 19.16  Media: 219.9  Inhibitor: 105.7 | Interaction: 0.0002  Media: <0.0001  Inhibitor: <0.0001 | Sidak’s multiple comparisons test | Normal  LPS + IL-4 vs Fluvastatin (24 hours): 0.0003  Fluvastatin vs GGPP (24 hours): 0.0042  LPS + IL-4 vs Fluvastatin (48 hours): <0.0001  Fluvastatin vs GGPP (48 hours): <0.0001 |  |
| 8J | Two-way ANOVA | Interaction: 96.40  Media: 66.99  Inhibitor: 388.8 | Interaction: <0.0001  Media: <0.0001  Inhibitor: <0.0001 | Sidak’s multiple comparisons test | CF  LPS + IL-4 vs Fluvastatin (24 hours): <0.0001  Fluvastatin vs GGPP (24 hours): >0.9999  LPS + IL-4 vs Fluvastatin (48 hours): <0.0001  Fluvastatin vs GGPP (48 hours): >0.9999 |  |

| Figure | Test | ANOVA  F value | ANOVA  P value | Test for multiple comparisons | Multiple comparisons (Adjusted P value) | Brown Forsythe test P value |
| --- | --- | --- | --- | --- | --- | --- |
| 9B | Two-way ANOVA | Interaction: 601.8  Generation: 1207  Treatment: 2.930 | Interaction: <0.0001  Generation: <0.0001  Treatment: 0.0218 | Sidak’s multiple comparisons test | Generation 1  Naïve vs LPS + IL-4: <0.0001  LPS + IL-4 vs PD18352: <0.0001  LPS + IL-4 vs VX745: 0.9950  LPS + IL-4 vs PD18352 + VX745: <0.0001  LPS + IL-4 vs Rapamycin: <0.0001  Generation 2  LPS + IL-4 vs PD18352: >0.9999  LPS + IL-4 vs VX745: 0.9238  LPS + IL-4 vs PD18352 + VX745: <0.0001  LPS + IL-4 vs Rapamycin: >0.9999  Generation 3  LPS + IL-4 vs PD18352: >0.9999  LPS + IL-4 vs VX745: 0.0134  LPS + IL-4 vs PD18352 + VX745: <0.0001  LPS + IL-4 vs Rapamycin: <0.0001  Generation 2  LPS + IL-4 vs PD18352: <0.0001  LPS + IL-4 vs VX745: <0.0001 |  |
| 9C | One-way ANOVA | 64.25 | <0.0001 | Dunnett’s multiple comparison’s test | Naïve vs LPS + IL-4: <0.0001  LPS + IL-4 vs PD18352: <0.0001  LPS + IL-4 vs VX745: 0.0409  LPS + IL-4 vs PD18352 + VX745: <0.0001  LPS + IL-4 vs Rapamycin: <0.0001 | 0.4358 |
| 9D | One-way ANOVA | 123.7 | <0.0001 | Dunnett’s multiple comparison’s test | Naïve vs LPS + IL-4: 0.0034  LPS + IL-4 vs PD18352: <0.0001  LPS + IL-4 vs VX745: >0.9999  LPS + IL-4 vs PD18352 + VX745: <0.0001  LPS + IL-4 vs Rapamycin: <0.0001 | 0.2771 |
| 9E | One-way ANOVA | 300.9 | <0.0001 | Dunnett’s multiple comparison’s test | Naïve vs LPS + IL-4: <0.0001  LPS + IL-4 vs PD18352: <0.0001  LPS + IL-4 vs VX745: 0.5399  LPS + IL-4 vs PD18352 + VX745: <0.0001  LPS + IL-4 vs Rapamycin: <0.0001 | 0.5122 |
| 9F | One-way ANOVA | 31.90 | <0.0001 | Dunnett’s multiple comparison’s test | Naïve vs LPS + IL-4: <0.0001  LPS + IL-4 vs PD18352: 0.0001  LPS + IL-4 vs VX745: 0.0013  LPS + IL-4 vs PD18352 + VX745: <0.0001  LPS + IL-4 vs Rapamycin: <0.0001 | 0.7256 |

| Figure | Test | ANOVA  F value | ANOVA  P value | Test for multiple comparisons | Multiple comparisons (Adjusted P value) | Brown Forsythe test P value |
| --- | --- | --- | --- | --- | --- | --- |
| 10A | Two-way ANOVA | Interaction: 14.67  Media: 66.83  Inhibitor: 711.2 | Interaction: <0.0001  Media: <0.0001  Inhibitor: <0.0001 | Tukey’s multiple comparisons test | Normal  Naïve vs IL-4:  <0.0001  Naïve vs LPS:  <0.0001  Naïve vs LPS + IL-4: <0.0001  Cholesterol-free  Naïve vs IL-4:  <0.0001  Naïve vs LPS:  <0.0001  Naïve vs LPS + IL-4: <0.0001 |  |
| 10B | One-way ANOVA | 284.4 | <0.0001 | Dunnett’s multiple comparison’s test | Naïve vs LPS: <0.0001  Naïve vs Resiquimod: <0.0001  Naïve vs CpG: <0.0001  Naïve vs Anti-IgM: <0.0001  Naïve vs CD40L: <0.0001 | 0.8282 |
| 10C | Two-way ANOVA | Interaction: 89.65  Inhibitor: 2014  Stimuli: 80.20 | Interaction: <0.0001  Inhibitor: <0.0001  Stimuli: <0.0001 | Tukey’s multiple comparisons test | Naïve vs LPS: 0.0178  LPS vs Fluvastatin: <0.0001  Naïve vs Anti-IgM: 0.9997  Anti-IgM vs Fluvastatin: <0.0001  Naïve vs CD40L: >0.9999  CD40L vs Fluvastatin: <0.0001  Naïve vs Resiquimod: 0.9975  Resiquimod vs Fluvastatin: <0.0001  Naïve vs CpG: 0.9396  CpG vs Fluvastatin: <0.0001 |  |
| 10E | Two-way ANOVA on log transformed data | Interaction: 94.68  Inhibitor: 1776  Stimuli: 40.29 | Interaction: <0.0001  Inhibitor: <0.0001  Stimuli: <0.0001 | Tukey’s multiple comparisons test | Naïve vs LPS: <0.0001  LPS vs Fluvastatin: <0.0001  Naïve vs Anti-IgM: 0.0535  Anti-IgM vs Fluvastatin: <0.0001  Naïve vs CD40L: >0.9999  CD40L vs Fluvastatin: <0.0001  Naïve vs Resiquimod: 0.0006  Resiquimod vs Fluvastatin: <0.0001  Naïve vs CpG: 0.0002  CpG vs Fluvastatin: <0.0001 |  |

| Figure | Test | ANOVA  F value | ANOVA  P value | Test for multiple comparisons | Multiple comparisons (Adjusted P value) | Brown Forsythe test P value |
| --- | --- | --- | --- | --- | --- | --- |
| Figure 1 – figure supplement 3 | One-way ANOVA | 71.82 | <0.0001 | Dunnett’s multiple comparison’s test | Naïve vs naïve + IFN-β: <0.0001  LPS vs LPS + IFN-β: <0.0001 | 0.3666 |

| Figure | Test | ANOVA  F value | ANOVA  P value | Test for multiple comparisons | Multiple comparisons (Adjusted P value) | Brown Forsythe test P value |
| --- | --- | --- | --- | --- | --- | --- |
| Figure 3 – figure supplement 1 | One-way ANOVA | 168.6 | <0.0001 | Dunnett’s multiple comparison’s test | Naïve vs LPS: <0.0001  Naïve vs Resiquimod: <0.0001  Naïve vs CpG: <0.0001  Naïve vs Anti-IgM: <0.0001  Naïve vs CD40L: <0.0001 | 0.7622 |

| Figure | Test | ANOVA  F value | ANOVA  P value | Test for multiple comparisons | Multiple comparisons (Adjusted P value) | Brown Forsythe test P value |
| --- | --- | --- | --- | --- | --- | --- |
| Figure 4 – figure supplement 2A | One-way ANOVA | 44.94 | <0.0001 | Dunnett’s multiple comparison’s test | Naïve vs LPS (2 hours): 0.0425  Naïve vs LPS (4 hours): <0.0001  Naïve vs LPS (8 hours): <0.0001 | 0.0846 |
| Figure 4 – figure supplement 2B | One-way ANOVA | 14.89 | <0.0001 | Dunnett’s multiple comparison’s test | Naïve vs LPS (2 hours): 0.2765  Naïve vs LPS (4 hours): 0.0019  Naïve vs LPS (8 hours): 0.0053 | 0.2086 |
| Figure 4 – figure supplement 2C | One-way ANOVA | 55.65 | <0.0001 | Dunnett’s multiple comparison’s test | Naïve vs LPS (2 hours): <0.0001  Naïve vs LPS (4 hours): <0.0001  Naïve vs LPS (8 hours): 0.0004 | 0.2816 |
| Figure 4 – figure supplement 2D | One-way ANOVA | 75.20 | <0.0001 | Dunnett’s multiple comparison’s test | Naïve vs LPS (2 hours): <0.0001  Naïve vs LPS (4 hours): <0.0001  Naïve vs LPS (8 hours): 0.0008 | 0.3293 |
| Figure 4 – figure supplement 2E | One-way ANOVA | 46.20 | <0.0001 | Dunnett’s multiple comparison’s test | Naïve vs LPS (2 hours): <0.0001  Naïve vs LPS (4 hours): <0.0001  Naïve vs LPS (8 hours): 0.1394 | 0.5125 |
| Figure 4 – figure supplement 2F | One-way ANOVA | 3.915 | 0.0319 | Dunnett’s multiple comparison’s test | Naïve vs LPS (2 hours): 0.2252  Naïve vs LPS (4 hours): 0.4463  Naïve vs LPS (8 hours): 0.3083 | 0.1257 |

| Figure | Test | ANOVA  F value | ANOVA  P value | Test for multiple comparisons | Multiple comparisons (Adjusted P value) | Brown Forsythe test P value |
| --- | --- | --- | --- | --- | --- | --- |
| Figure 5 – figure supplement 2A | One-way ANOVA | 110.1 | <0.0001 | Dunnett’s multiple comparison’s test | LPS + IL-4 vs 0.1µM Fluvastatin: >0.9999  LPS + IL-4 vs 1µM Fluvastatin: 0.6755  LPS + IL-4 vs 10µM Fluvastatin: 0.0642  LPS + IL-4 vs 50µM Fluvastatin: <0.0001  LPS + IL-4 vs 100µM Fluvastatin: <0.0001 | 0.2767 |
| Figure 5 – figure supplement 2B | One-way ANOVA | 24.24 | <0.0001 | Dunnett’s multiple comparison’s test | LPS + IL-4 vs 0.1µM Fluvastatin: 0.3002  LPS + IL-4 vs 1µM Fluvastatin: 0.1048  LPS + IL-4 vs 10µM Fluvastatin: 0.0055  LPS + IL-4 vs 50µM Fluvastatin: 0.0041  LPS + IL-4 vs 100µM Fluvastatin: 0.9990 | 0.5532 |
| Figure 5 – figure supplement 2D | One-way ANOVA | 18.37 | <0.0001 | Dunnett’s multiple comparison’s test | LPS + IL-4 vs 0.1µM  NB-598: 0.9921  LPS + IL-4 vs 1µM  NB-598: 0.9641  LPS + IL-4 vs 10µM  NB-598: 0.0215  LPS + IL-4 vs 50µM  NB-598: 0.0016  LPS + IL-4 vs 100µM  NB-598: <0.0001 | 0.1551 |
| Figure 5 – figure supplement 2E | One-way ANOVA | 35.70 | <0.0001 | Dunnett’s multiple comparison’s test | LPS + IL-4 vs 0.1µM  NB-598: 0.5711  LPS + IL-4 vs 1µM  NB-598: 0. 0384  LPS + IL-4 vs 10µM  NB-598: 0.2167  LPS + IL-4 vs 50µM  NB-598: 0.0727  LPS + IL-4 vs 100µM  NB-598: 0.0003 | 0.4192 |
| Figure 5 – figure supplement 2G | One-way ANOVA | 80.33 | <0.0001 | Dunnett’s multiple comparison’s test | LPS + IL-4 vs 0.1µM Rosuvastatin: 0.0078  LPS + IL-4 vs 1µM Rosuvastatin: 0.0034  LPS + IL-4 vs 10µM Rosuvastatin: <0.0001  LPS + IL-4 vs 50µM Rosuvastatin: <0.0001  LPS + IL-4 vs 100µM Rosuvastatin: <0.0001 | 0.8511 |
| Figure 5 – figure supplement 2H | One-way ANOVA | 37.33 | <0.0001 | Dunnett’s multiple comparison’s test | LPS + IL-4 vs 0.1µM Rosuvastatin: 0.4964  LPS + IL-4 vs 1µM Rosuvastatin: 0.5414  LPS + IL-4 vs 10µM Rosuvastatin: 0.0767  LPS + IL-4 vs 50µM Rosuvastatin: 0.0052  LPS + IL-4 vs 100µM Rosuvastatin: 0.0005 | 0.2395 |
| Figure 5 – figure supplement 2J | One-way ANOVA | 197.4 | <0.0001 | Dunnett’s multiple comparison’s test | LPS + IL-4 vs 0.1µM Rosuvastatin: 0.0039  LPS + IL-4 vs 1µM Rosuvastatin: <0.0001  LPS + IL-4 vs 10µM Rosuvastatin: <0.0001  LPS + IL-4 vs 50µM Rosuvastatin: <0.0001  LPS + IL-4 vs 100µM Rosuvastatin: <0.0001 | 0.5381 |

| Figure | Test | ANOVA  F value | ANOVA  P value | Test for multiple comparisons | Multiple comparisons (Adjusted P value) | Brown Forsythe test P value |
| --- | --- | --- | --- | --- | --- | --- |
| Figure 6 – figure supplement 1G | One-way ANOVA | 31.18 | <0.0001 | Dunnett’s multiple comparison’s test | LPS + IL-4 vs Fluvastatin (normal): 0.0003  LPS + IL-4 vs LPS + IL-4 (normal/CF): 0.0047  LPS + IL-4 vs Fluvastatin (CF): <0.0001 | 0.2643 |
| Figure 6 – figure supplement 1H | One-way ANOVA | 20.61 | <0.0001 | Dunnett’s multiple comparison’s test | LPS + IL-4 vs Fluvastatin (normal): 0.0022  LPS + IL-4 vs LPS + IL-4 (normal/CF): 0.0066  LPS + IL-4 vs Fluvastatin (CF): <0.0001 | 0.1823 |
| Figure 6 – figure supplement 1I | One-way ANOVA | 55.50 | <0.0001 | Dunnett’s multiple comparison’s test | LPS + IL-4 vs Fluvastatin (normal): 0.0006  LPS + IL-4 vs LPS + IL-4 (normal/CF): <0.0001  LPS + IL-4 vs Fluvastatin (CF): <0.0001 | 0.1322 |

| Figure | Test | ANOVA  F value | ANOVA  P value | Test for multiple comparisons | Multiple comparisons (Adjusted P value) | Brown Forsythe test P value |
| --- | --- | --- | --- | --- | --- | --- |
| Figure 7 – figure supplement 1A | One-way ANOVA | 109.2 | <0.0001 | Dunnett’s multiple comparison’s test | Naïve vs LPS + IL-4: <0.0001  LPS + IL-4 vs 1mM MVA: 0.9879  LPS + IL-4 vs 2mM MVA: 0.2461  LPS + IL-4 vs 4mM MVA: <0.0001 | 0.6727 |
| Figure 7 – figure supplement 1B | One-way ANOVA | 105.7 | <0.0001 | Dunnett’s multiple comparison’s test | Naïve vs LPS + IL-4: 0.0187  LPS + IL-4 vs 1mM MVA: 0.0625  LPS + IL-4 vs 2mM MVA: <0.0001  LPS + IL-4 vs 4mM MVA: <0.0001 | 0.3874 |

| Figure | Test | ANOVA  F value | ANOVA  P value | Test for multiple comparisons | Multiple comparisons (Adjusted P value) | Brown Forsythe test P value |
| --- | --- | --- | --- | --- | --- | --- |
| Figure 9 – figure supplement 2A | One-way ANOVA | 165.6 | <0.0001 | Dunnett’s multiple comparison’s test | Naïve vs LPS + IL-4: <0.0001  LPS + IL-4 vs Rapamycin: 0.0137 | 0.6914 |
| Figure 9 – figure supplement 2B | One-way ANOVA | 345.0 | <0.0001 | Dunnett’s multiple comparison’s test | Naïve vs LPS + IL-4: <0.0001  LPS + IL-4 vs Rapamycin:  0.0001 | 0.2597 |
